# Mixed Hepatoblastoma in a Young Male Adult: A Case Report and Literature Review

**DOI:** 10.1155/2010/919457

**Published:** 2010-11-22

**Authors:** Valeria Fiaschetti, Roberto Fiori, Eleonora Gaspari, Sonia Crusco, Giovanni Simonetti

**Affiliations:** Dipartimento di Diagnostica per Immagini, Imaging Molecolare, Radiologia Interventistica e Radioterapia, Policlinico Universitario “Tor Vergata”, Viale Oxford 81, 00133 Roma, Italy

## Abstract

Hepatoblastoma (HB) is a rare malignant tumour of the liver and usually occurs in the first three years of life. Most of these tumours arise in the embryo; hence it seems to be unusual that hepatoblastoma occurs in adults and is an exceptional cause of primary malignant liver tumour in adult patients. The diagnosis is often overlooked, and patients might be diagnosed at late stages of the disease at risk of increased mortality. In this paper we report a case of a 30-year-old man with mixed hepatoblastoma and abdominal pain, hepatomegaly and fever. The patient under went noninvasive diagnostic methods: duplex scanning, Computed Tomography (CT), and Magnetic Resonance imaging (MRI). In our experience, despite the important role of histological sample provided by biopsy in defining the diagnosis, very important was the role of MRI, more than ultrasonography and enhanced CT. The MRI detects tumor features as size, margins, and ratio with neighboring organs in order to get the best surgical approach.

## 1. Introduction

Epithelial carcinomas of liver are among the most frequent malignant tumours that occur in adults [1]. Far more than 90% represent either hepatocellular carcinoma (HCC) or intrahepatic cholangiocarcinoma. Rare malignancies in adults with epithelial differentiation include combined hepatocellular or cholangiocarcinoma, carcinosarcoma, oval or stem-cell neoplasm, and hepatoblastoma [2].

Hepatoblastoma (HB) is a rare malignant tumour of the liver and usually occurs in the first three years of life [3]. Most of these tumours arise in the embryo; hence it seems to be unusual that hepatoblastomas occur in adults and are an exceptional cause of primary malignant liver tumour in adult patients [4]. Various synonymous have been used to describe this kind of tumour such as hepatic embryonic mixed tumour, rhabdomyosarcohepatoma, carcino-osteochondromyxosarcoma, and malignant mixed hepatoblastoma [5].

However, the existence of HB in adult patients has been refuted by some authors, with nonspecific initial symptoms and difficulty in discerning abnormalities in laboratory data of the patients [6, 7]. Consequently, the diagnosis is often overlooked, and patients might be attended at a late stage of the disease, at risk of increased mortality.

In this paper we report a case of mixed hepatoblastoma, diagnosed in our department of diagnostic imaging, in a young adult patient with abdominal pain, hepatomegaly, and fever.

## 2. Case Report

A 30-year-old man had been suffering from right hypochondriac pain and fever. He had no history of the disease and had not received blood transfusion. Furthermore, there was not family history of liver disease. Physical examination revealed that the liver was palpable 5 cm below the right costal margin. 

Laboratory data showed the following values: aspartate aminotransferase (AST) 23 IU/L; alanine aminotransferase (ALT) 18 IU/L; total cholesterol 154 mg/dL; negative hepatitis B surface antigen, antihepatitis B antibody, and antihepatitis C antibody; alpha-fetoprotein (AFP) 45 ng/mL and total bilirubina 1,71 mg/dL, PCR 54, 50 mg/L (0–5), VES 55 mm/h (2–25). There was no evidence of liver cirrhosis. Blood cultures and screening for tuberculosis were negative.

The patient under went noninvasive diagnostic methods: duplex scanning, Computed tomography (CT) and Magnetic Resonance imaging (MRI).

**Figure 1:**
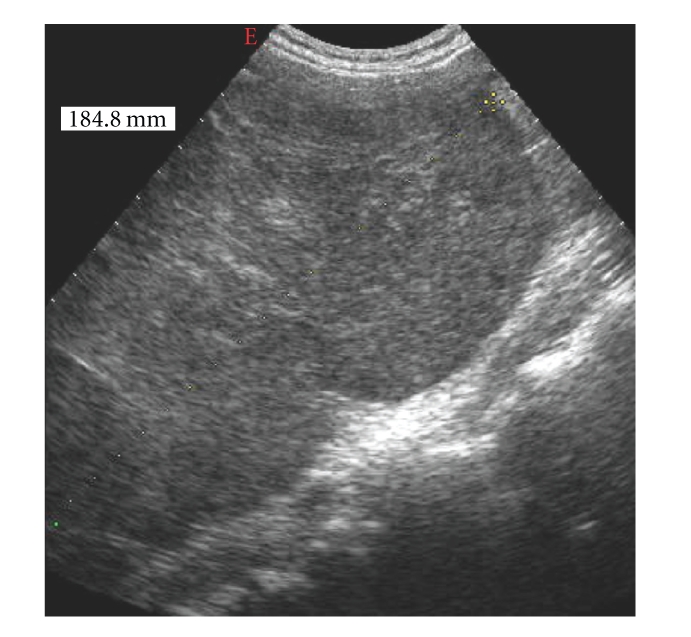
The Duplex scanning showed hepatomegaly (18 cm of longitudinal diameter) with presence of heterogeneous mass in the right lobe.

The Duplex scanning showed hepatomegaly (18 cm of longitudinal diameter) with presence of heterogeneous mass in the right lobe. The mass was hyperechoic with some calcifications and few anechoic foci, secondary to haemorrhage and necrotic processes (Figure 1).

The color Doppler ultrasonography (US-CD) demonstrated normal patency of sovrahepatic vessels.

Subsequently the patient was submitted to Computed Tomography (CT) of abdomen (scanner : Highspeed Advantage; GE Medical System Milwaukee, WI, USA). 

Images were acquired without and with an intravenously contrast media (a single bolus of 120 cc with a flow rate of 2.5 mL/s of nonionic contrast medium via an antecubital venous access), and scans were obtained with threephases from the injection of contrast media. 

**Figure 2:**
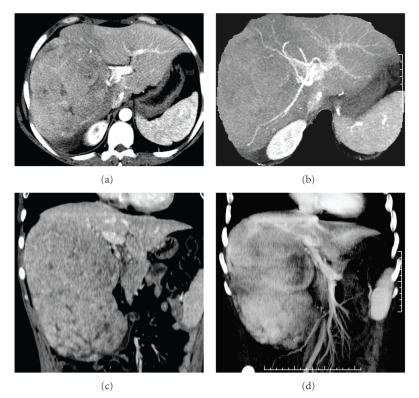
Computed Tomography (CT): axial arterial phase (a); coronal venous phase (c); MIP axial and coronal (b–d). The CT confirmed a mass with heterogeneous density that occupied almost the whole right lobe of the liver (a–c). MIP images showed compressive effects of the mass on sovrahepatic veins; the vessels, were displaced but not infiltrated (b–d).

The CT confirmed a mass that occupied almost the whole right lobe of the liver with lower attenuation than surrounding liver on nonenhanced scan. After the injection of the contrast media, it was assessed that the lesion was predominantly of lower attenuation with some calcifications and small slit like or round and lower density areas, corresponding to haemorrhage or necrosis. Contrast enhancement was low and heterogeneous (Figure 2).

**Figure 3:**
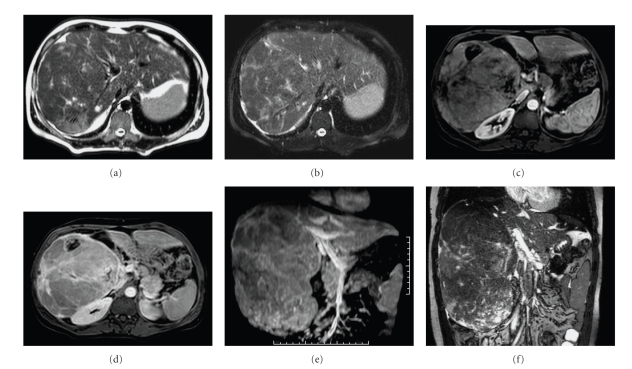
Magnetic Resonance Imaging (MRI): axial T2-weighted images (a); axial T2-SPAIR weighted image (b); axial and coronal THRIVE (c–e); coronal balanced (f); T2-weighted images demonstrated a 23 × 14 × 13 cm heterogeneous mass in the right lobe (a-b). THRIVE images showed heterogeneous contrast enhanced with enhancement of fibrosis bands in tardive-phase (c–e). This heterogeneous mass occupied almost the whole right lobe of the liver presenting a well-defined capsule (f).

Magnetic Resonance imaging (MRI) with contrast media demonstrated a 23 × 14 × 13 cm mass in the right lobe. This mass presented a well-defined capsule. T1-weighted images showed heterogeneous global intensity with areas with intensity signal similar to haemoglobin metabolites, areas with signal intensity similar to calcifications and area with signal intensity similar to fat. On T2-weighted images the lesion was hyperintense but heterogeneous because of hypointense areas corresponding to calcifications and bands corresponding to area of lobulation and fibrosis. Furthermore right kidney was displaced posteriorly by the mass with its vascular pedicle (Figure 3).

Compressive effects of the mass on sovrahepatic veins were observed without occlusion, while gallbladder was displaced but not infiltrated. 

Related to MRI features was supposed a hepatoblastic nature of the lesion.

**Figure 4:**
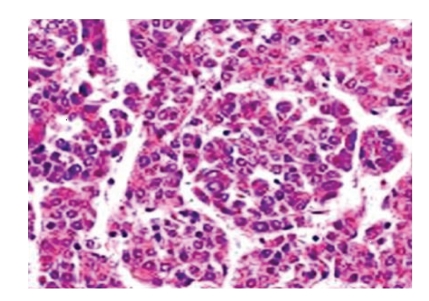
Microscopic examination of mass was composed by a combination of mesenchymal and epithelial elements. The surgical diagnosis confirmed the mixed hepatoblastoma.

Subsequent diagnostic imaging phase, a bioptic sample was obtained with a CT-guided biopsy with 18 G tru-cut needle. Histopathologic examination revealed the presence of mesenchymal and epithelial cells in bioptic specimen and confirmed the diagnosis of a mixed hepatoblastoma (Figure 4).

After results of biopsy, surgical intervention was performed in exploratory to resect the tumour. A right trisegmentectomy was performed with tumour grossly resected with microscopic residual disease. The surgical diagnosis confirmed the diagnosis of a mixed hepatoblastoma. 

No postprocedural complications were observed. He is receiving the VI cycles of systemic chemotherapy whit adriamycin and cisplatin which results in good health.

## 3. Discussion

HB is the most common primary malignant liver neoplasm in children [8, 9]. Approximately 90% of the cases occur in patients under 5 years of age, and two thirds of the cases occur in the first 2 years of life [10, 11]. HB in adolescent and young adults is extremely rare nevertheless the prognosis is much worse than in childhood, because these kind of tumours are usually diagnosed late [4, 12]. Some studies have shown a male to female ratio for hepatoblastoma patients of 3-2 : 1 [13].

The etiology of HB has been elusive. Present investigations of the cytogenetic and molecular genetic abnormalities in HB revealed involvement of chromosomal loci on 1q, 2 (or 2q), 4q, 8 (or 8q), and 20. Loss of heterozygosity imprinting at locus 11p 15.5 also suggests a common genetic basis for HB [14]. The detection of nuclear *β*-catenin accumulation implies an oncogene alteration of the wnt/*β*-catenin pathway. Furthermore, nuclear p53 accumulation indicates that p53 mutation is also involved in the molecular pathogenesis of the malignancy [15]. Based on embryological theory, it is believed that HB arises from a hepatic blastema. However, this hypothesis seems to be inapplicable to adult HB. Only four patients [6, 16, 17] in the literature were more than 70 years old. The persistence of primitive hepatic blastema for such a long period seems unlikely. Furthermore, the presence of cirrhosis in liver with HB is not seen in children. 

However, cirrhosis of the liver has been seen in association with adult HB in 30% of cases [6, 18]. In 20%–30% of the cases, there are calcifications in the mixed HB.

This would imply that these tumours may have a different pathogenetic pathway in adults compared to children. 

Ishank and Glunz classified hepatoblastomas into two groups: epithelial type and mixed epithelial and mesenchymal type [19].

The epithelial type consists of fetal and embryonic cells presenting alone or in combination; in the epithelio-mesenchymal mixed type, mesenchymal elements are present along with the epithelial component [20]. For a long time it has been thought that hepatoblastoma develops during intrauterine life, but the same histological pattern has been seen in hepatic tumours in adults, and new data about its histogenesis are emerging. An interesting theory looks at the common hepatocytes as the starting point, after having lost the differentiation and acquired new possibilities of transformation [21]. 

In adults, the morbidity of HB is extremely rare, and the initial symptoms are nonspecific so that the diagnosis is often overlooked. The usual presentation is failure to *thrive*, loss of weight, and a rapidly enlarging upper abdominal mass. The serum AFP level is almost invariably high [22], such as in our case. 

The initial diagnosis of HB is mainly based on imaging. Proper diagnosis, staging, and treatment of HB require accurate imaging studies. Ultrasound (US) is a noninvasive modality that is particularly useful in the evaluation of infants. HB is seen as a hyperechoic, solid, intrahepatic mass on US [23]. Other standard investigations include Computed tomography (CT), Magnetic Resonance imaging (MRI), and serum AFP. However, the final diagnosis relies on tumour biopsy. Furthermore, it is also difficult to make a pathologic diagnosis of adult HB, since there are several similar types of tumours such as hepatic teratoma, carcinosarcoma, malignant mesenchymal tumor, and HCC with sarcomatous changes and hepatoblastomatous lesions [16].

The complete surgical resection is the cornerstone of treatment for patients with HB and is the only chance of an optimal clinical result. Despite this, the improvements in survival that have occurred over the last three decades have been the function of standardized chemotherapy that reduces tumor size and enables complete tumor excision, even permitting cure in the presence of initially unresectable or metastatic disease [24]. Chemotherapy has been proven effective in both an adjuvant and neoadjuvant treatment and can shrink tumors. It makes them less prone to bleed and delineates the tumor from the surrounding normal parenchyma and vascular structures so as to facilitate the resections. HB is sensitive to such chemotherapy drugs as doxorubicin, cisplatin, vincristine, 5-FU, and cyclophosphamide [25]. 

In our experience, despite the important role of histological sample provided by biopsy in defining diagnosis, very important is the role of MRI, more than ultrasonography and enhanced CT, in depicting tumor features as size, margins, and ratio with neighboring organs in order to the best surgical approach.
